# Polycarboxy/Sulfo Betaine—Calcium Phosphate Hybrid Materials with a Remineralization Potential

**DOI:** 10.3390/ma16206640

**Published:** 2023-10-11

**Authors:** Diana Rabadjieva, Rumiana Gergulova, Konstans Ruseva, Alexander Bonchev, Pavletta Shestakova, Marin Simeonov, Radosveta Vasileva, Dragomir Tatchev, Rositsa Titorenkova, Elena Vassileva

**Affiliations:** 1 Institute of General and Inorganic Chemistry, Bulgarian Academy of Sciences, Acad. G. Bonchev Str., bl. 11, 1113 Sofia, Bulgaria; rumigg@yahoo.com; 2 Laboratory on Structure and Properties of Polymers, Faculty of Chemistry and Pharmacy, University of Sofia, 1, James Bourchier Blvd., 1164 Sofia, Bulgaria; ohtkr@chem.uni-sofia.bg (K.R.); m.simeonov@chem.uni-sofia.bg (M.S.); ohtev@chem.uni-sofia.bg (E.V.); 3 Faculty of Dental Medicine, Medical University, 1, G. Sofiiski Str., 1431 Sofia, Bulgaria; dralexanderbonchev@abv.bg (A.B.); etienet@abv.bg (R.V.); 4 Institute of Organic Chemistry with Centre of Phytochemistry, Bulgarian Academy of Sciences, Acad. G. Bonchev Str., bl. 9, 1113 Sofia, Bulgaria; pavletta.shestakova@orgchm.bas.bg; 5 Rostislaw Kaischew Institute of Physical Chemistry (IPC), Bulgarian Academy of Sciences, Acad. G. Bonchev Str., bl. 11, 1113 Sofia, Bulgaria; dtachev@ipc.bas.bg; 6 Institute of Mineralogy and Crystallography, Bulgarian Academy of Sciences, Acad. G. Bonchev Str., bl. 107, 1113 Sofia, Bulgaria; rositsatitorenkova@imc.bas.bg

**Keywords:** biomimetic synthesis, de- and remineralization, micro-CT analysis, NMR, pre-nucleation clusters, zwitterionic functionality

## Abstract

Biomacromolecules control mineral formation during the biomineralization process, but the effects of the organic components’ functionality on the type of mineral phase is still unclear. The biomimetic precipitation of calcium phosphates in a physiological medium containing either polycarboxybetaine (PCB) or polysulfobetaine (PSB) was investigated in this study. Amorphous calcium phosphate (ACP) or a mixture of octacalcium phosphate (OCP) and dicalcium phosphate dihydrate (DCPD) in different ratios were identified depending on the sequence of initial solution mixing and on the type of the negative functional group of the polymer used. The more acidic character of the sulfo group in PSB than the carboxy one in PCB determines the dominance of the acidic solid phases, namely, an acidic amorphous phase or DCPD. In the presence of PCB, the formation of ACP with acicular particles arranged in bundles with the same orientation was observed. A preliminary study on the remineralization potential of the hybrid material with the participation of PSB and a mixture of OCP and DCPD did not show an increase in enamel density, contrary to the materials based on PCB and ACP. Moreover, the latter showed the creation of a newly formed crystal layer similar to that of the underlying enamel. This defines PCB/ACP as a promising material for enamel remineralization.

## 1. Introduction

The preparation of inorganic/organic hybrid materials (HMs) via a macromolecule-controlled biomimetic process helps for understanding naturally occurring biomineralization and results in the development of novel hybrid biomaterials with potential applications in dental medicine.

Biomineralization is a process by which inorganic mineral crystals are formed and deposited in an organic matrix. Thus, structures with unique physicochemical and mechanical properties are formed. The organic substances involved in the process control the nucleation, orientation, and bonding of the newly formed nuclei into crystalline formations [1]. The proteins amelogenin, enamelin, ameloblastin, and amelotin together with matrix metalloprotease-20 and kallikrein-4 are responsible for dental enamel formation [1,2], whose mineral part is hydroxyapatite. Amelogenin is a major organic component. Its composition is rich in histidine, glutamine, proline, and leucine amino acid residues, which are responsible for protein–mineral interactions [3,4]. Studying the murine tooth enamel, Beniash et al. [5] reported that, at the beginning of the enamel genesis, mineral particles from amorphous calcium phosphate, suspended in a protein gel, are formed, which furthermore transform into apatite crystals. Tao et al. [4] found that the adsorption of amelogenin onto mineral surfaces depends on the amelogenin structure and affects the induction time for the transformation of the amorphous calcium phosphate to hydroxyapatite. The absence of amelogenin and metalloprotease-20 led to the formation of octacalcium phosphate instead of amorphous calcium phosphate [2]. All studies on the mechanism of enamel formation reveal the leading role of bio-macromolecules, which shows the potential of composite and hybrid organic/inorganic materials as remineralizing agents.

Hybrid materials are intriguing for a variety of applications due to their properties, which are a result of the synergetic effect of the organic and inorganic components’ combination. Polymer-based biocompatible materials have attracted a lot of interest due to the possibility of using them to protect human health and improve the quality of life [6]. As inorganic components’ different substances like silanized silica particles [7], graphene/carbon nanotubes [8], bioactive ceramics, and glasses or glass ceramics [9], metallic materials [10], etc., have been used depending on the biomaterial application. Calcium phosphate/polymer hybrid materials are preferred for the remineralization of primary tooth enamel defects as they promote fast and noninvasive enamel restoration [11]. Although hydroxyapatite (HA) is considered the closest in composition and structure to the mineral part of enamel, amorphous calcium phosphate (ACP) and α- or β-tricalcium phosphate (TCP) are usually used in hybrid remineralization systems. ACP is a primary solid phase precipitated from calcium phosphate solutions at physiological pH. It is a metastable phase and transforms into thermodynamically stable HA with time. In addition, the solubility of ACP is higher than that of β-TCP and even of α-TCP, which accelerates the dissolution/crystallization/recrystallization processes [12]. 

Polysaccharides as cellulose [13], different synthetic polymers [14,15] (polylactic acid, poly(lactic-co-glycolic acid), polycaprolactone, etc.), and natural substances [16,17,18] (collagen, chitosan, gelatine, etc.) have been used as organic components in HMs. Investigations concern mainly the effect of the preparation method and the type of organic compounds on the mechanical properties and the bioactivity of the obtained biomaterials [19,20]. Jee et al. [21] showed that the molecular weight of poly-L-aspartic acid influenced the degree of the mineralization process. Amino acids were also used in a large part of the investigations because they simulate the behavior of the corresponding residues in the protein structure [22,23,24]. Hybrid materials are usually prepared by in situ precipitation of calcium phosphate (CaP) in the presence of amino acids. The effect of the amino acid side chain charge on the structure, morphology, and surface properties of apatite crystals have been studied.

Synthetic polymers offer a variety of functional groups, which could be identical to the ones possessed by the natural polymers or scaffolds, but at the same time, they offer other parameters that could control the mineralization process, e.g., template architecture, topology, different molecular weights, self-organization, as well as some additional benefits. Polyzwitterions are a unique class of synthetic polymers as they possess both positively and negatively charged functional groups, which are directly covalently bonded to each other and provide a functionality analogous to the naturally occurring one in the betaine form of amino acids, in the polar heads of phospholipids, etc. Polyzwitterions, in particular PSB and PCB, have an expanding popularity in biomedical applications due to their high biocompatibility, “smart” behavior, as they are able to respond to variations in pH, temperature, salt concentration, as well as to their antibiofouling behavior [25,26,27]. The latter has been exploited recently for dental applications [28], but still the advantages of their zwitterionic structure for controlled calcium phosphates’ mineralization has not been clearly explored. PCB and PSB, in their side chains, bear negatively charged acidic end groups, respectively, carboxy or sulfo, which are expected to interact differently with Ca^2+^ ions supplied for the mineralization process. Thus, PSB and PCB could not only control calcium phosphate formation but would also bear antibiofouling activity, which is especially beneficial for dental materials.

Despite the accumulated data, the relationship between the functionality of the organic components used and the resulting hybrid calcium phosphate systems is still unclear.

The aim of the present work is to study the biomimetic precipitation of calcium phosphates in a physiological medium containing polymer with betaine functionality, either polycarboxybetaine (PCB) or polysulfobetaine (PSB), in order to (i) elucidate the effect of betaine moieties and the synthesis route on the phase composition of calcium phosphates and (ii) prepare and characterize new hybrid materials with a potential remineralization effect. Two synthesis routes, differing in the mixing sequence of the starting solutions, were studied in order to elucidate the effect of the initial pH and of the dominant ions on the type of the pre-nucleation clusters and thus on the solid phases formed. The changes in pH and in the concentration of Ca^2+^ ions during the syntheses were followed in three types of systems—a polymer-free system, a system with PSB, and a system with PCB—to explore the effects of betaine zwitterionic functionality and to prepare different types of HMs. The materials, which were prepared for the first time, were characterized by XRD, NMR, TEM, and DTA-TG-MASS analyses. Further, a preliminary study on the remineralization potential of the selected materials was carried out, and the results were proof by micro-CT, IR, and SEM analyses.

## 2. Materials and Methods

### 2.1. Synthesis of Polymers 

#### 2.1.1. RAFT Polymerization of Carboxybetaine (CB) Monomers

In a 250 mL round-bottom flask, 10 g of CB monomers, obtained according to a procedure described elsewhere [29] (Scheme S1, Supplementary Materials), and 0.1 mol. % 2,2-azobis(2-methylpropionamide) dihydrochloride were dissolved in a 45 mL acetate buffer (pH = 5.2, 0.27 mol·L^−1^ acetic acid and 0.73 mol·L^−1^ sodium acetate). A total of 0.01 mol. % 4-cyano-4-(thiobenzoylthio)pentanoic acid (CTPA, Sigma Aldrich, St. Louis, MO, USA, A.R.) was neutralized in 5 mL of 0.05 M KOH (Sigma Aldrich, St. Louis, MO, USA, A.R.) and then added dropwise to the CB monomer solution. The amount of CTPA was calculated in such a way as to give a linear PCB with a molar mass of 100,000 g/mol (Scheme S1, Supplementary Material). The solution was heated for 5 h at 70 °C and then placed in an ice bath to stop the polymerization process. PCB was dialyzed against distilled water for two weeks using the dialysis membrane 3.5 K MWCO, 16 mm, SnakeSkin™ Dialysis Tubing, Thermo Scientific, Waltham, MA, USA, until no more traces of residual monomers and other reactants were detected in the wastewater (these were monitored with UV/Vis (JAsco V-730, Jasco, Tokyo, Japan)). The resulting polymer was freeze-dried, thus obtaining a white powder. The conversion was determined gravimetrically to be 87%. The successful polymerization and the chemical structure of the polymer was confirmed by ^1^H NMR spectroscopy (Figure S1 in the Supplementary Materials). 

#### 2.1.2. RAFT Polymerization of Sulfobetaine (SB) Monomers

The RAFT polymerization of sulfobetaine monomers was carried out following Scheme S2 in the Supplementary Materials. A total of 5 g of the monomer sulfobetaine methacrylate (SB, Sigma-Aldrich, St. Louis, MO, USA, A.R.) was dissolved in a 20 mL acetate buffer with a pH of 5.2. Initiator 2,2-azobis(2-methylpropionamide) dihydrochloride (Sigma-Aldrich, St. Louis, MO, USA, A.R.) with a concentration of 0.1 mol was added, with respect to the amount of the SB monomer. A total of 14 mg of CTPA was dissolved in 5 mL of 0.05 M NaOH (Sigma-Aldrich, St. Louis, MO, USA, A.R.), and the resulting solution was added dropwise to the reaction mixture. The amount of CTPA was calculated to give a linear PSB with a molar mass of 100,000 g/mol (Scheme S2, Supplementary Materials). The solution was heated for 5 h at 70 °C and then placed in an ice bath to stop the polymerization process. PSB was dialyzed against distilled water for two weeks using the dialysis membrane 3.5 K MWCO, 16 mm, SnakeSkin™ Dialysis Tubing, Thermo Scientific, Waltham, MA, USA, until no more traces of residual monomers and other reagents were detected in the wastewater (these were monitored with UV/Vis (JASCO V-730, Jasco, Japan)). The resulting polymer was freeze-dried, thus obtaining a white powder. The conversion was determined gravimetrically to be 73%. The successful polymerization and the chemical structure of the polymer was confirmed by ^1^H NMR spectroscopy (Figure S2 in the Supplementary Materials).

### 2.2. Biomimetic Precipitation of Calcium Phosphates in a Physiological Medium Containing Polymers with Betaine Functionality

A combined apparatus for automatic titration and controlled synthesis (Titrando 907, Methrom AG, Herisau, Switzerland) was used to maintain a constant flow rate and to monitor the pH and concentration of free Ca^2+^ ions during the synthesis procedures. The initial substances CaCl_2_.2H_2_O (Sigma-Aldrich, St. Louis, MO, USA, A.R.) and Na_2_HPO_4_ (Merck, Darmstadt, Germany, A.R.) were dissolved in a physiological solution (0.9% NaCl, Merck, Darmstadt, Germany, A.R.) to obtain 0.05 M of a Ca solution and 0.03 M of a P solution, respectively. PCB or PSB, with a molar ratio (monomeric unit)/Ca^2+^ of 1, were dissolved in the Ca solution. An experiment without polymers was also performed for the sake of a comparison. Two series of experiments were carried out, namely, the following:

Series A: A total of 130 mL of the Ca solution was added to 130 mL of the P solution. The products of this series will be called further as follows: A-CaP—for a polymer-free system, A-PSB/CaP—for a system with PSB, and A-CaP/PCB—for a system with PCB.
Series B: A total of 130 mL of the P solution was added to 130 mL of the Ca solution. The products of this series will be called further as follows: B-CaP—for a polymer-free system, B-PSB/CaP—for a system with PSB, and B-CaP/PCB—for a system with PCB.

The parameters of the process are an addition rate of 3 mL/min (0.25 mL per step), the room temperature, constant stirring, and a Ca/P molar ratio of 1.67, which is characteristic for hydroxyapatite. 

The pH and concentration of the free Ca^2+^ ions were monitored using combined pH (iConneet, Methrom AG, Herisau, Switzerland) and Ca^2+^ polymer membrane ion selective electrodes (Methrom AG, Herisau, Switzerland). A calibration standard curve for calculating free Ca^2+^ ion concentrations was generated by a measurement of the electrical potential of the standard solutions of CaCl_2_ in 0.9% solution of NaCl. The concentrations of the standard solutions were within the concentration range of the Ca^2+^ ions under the experimental conditions. 

The obtained suspension was left to mature for 1 h, washed out from Na^+^ and Cl^−^ residuals using a dialysis tube (3.5 K MWCO, 16 mm, SnakeSkin™ Dialysis Tubing, Thermo Scientific, Waltham, MA, USA), and then freeze-dried. 

### 2.3. Characterization

#### 2.3.1. Powder X-ray Diffraction Analysis

A Bruker D8 Advance diffractometer with Cu K radiation and a LynxEye detector (Bruker AXS Advanced X-ray Solutions GmbH, Karlsruhe, Germany) was used to conduct powder X-ray diffraction. The data were gathered in the 10 to 90° 2θ range with a step of 0.03° 2θ and a counting rate of 57 s/step for the primary phase identification. The phase composition was identified with the ICSD database.

#### 2.3.2. Solid State Nuclear Magnetic Resonance (NMR) Analysis 

NMR spectra were recorded on a Bruker Avance II+ 600 spectrometer (^1^H working frequency of 600.01 MHz and 242.94 MHz for ^31^P), equipped with a 4 mm ^1^H/^31^P-15N solid state CP MAS dual ^1^H/X probe head. The samples were loaded in 4 mm ZrO_2_ rotors and spun at a magic angle spinning (MAS) rate of 10 kHz for all measurements. The quantitative ^31^P NMR spectra were acquired with a single pulse sequence (Bruker Topspin library), with a 90° pulse length of 3.1 µs, 8 K time domain data points, a spectrum width of 74 kHz, 256 scans, and a relaxation delay of 120 s. The spectra were processed with an exponential window function (line broadening factor 5) and zero-filled to 32 K data points. The ^1^H→^31^P cross-polarization MAS (CP-MAS) spectra were acquired with the following experimental parameters: a 1H excitation pulse of 3.4 μs, 10 s of a relaxation delay, 256 scans, and a MAS rate of 12 kHz. A series of spectra with a contact time varied from 100 µs up to 5 ms were measured. A ^1^H SPINAL-64 decoupling scheme was used during the acquisition of the CP experiments. All ^31^P chemical shifts were referenced against the external solid reference NH_4_H_2_PO_4_ (δ 0.9 ppm). The DMfit software (release #20220502)was used for the deconvolution, simulation, and fitting of the experimental NMR data [30]. The ^1^H NMR spectra of PSB and PCB were measured on the same spectrometer, using a 5 mm direct detection dual probe head (BBO) ^31^P–^109^Ag/^1^H with a gradient coil with a maximum gradient strength of 53 G/cm.

#### 2.3.3. Differential Thermal Analysis with Release Gas Detection (DTA-TG-MASS Analysis)

The thermal characterization of the samples was performed on a LABSYS^TM^ EVO (Setaram, Caluire, France) apparatus with a Pt/Pt-Rh thermocouple in a corundum crucible at a heating rate of 10 °C/minute in a temperature range of 25–900 °C and in an atmosphere of synthetic air. This apparatus was equipped with a quadrupole mass spectrometer (Pfeiffer vacuum OMNISTAR, GSD 301, Zürich, Switzerland), which serves for the analysis of the escaping gasses. In the studied samples, the release of H_2_O (m = 18) and CO_2_ (m = 44) was determined.

#### 2.3.4. Transmission Electron Microscopy (TEM)

The morphology of the selected samples was examined by transmission electron microscopy (JEOL transmission electron microscope, JEM-2100, Tokyo, Japan), equipped with an EDS detector (X-Max 80T, Oxford Instruments, High Wycombe, UK). Samples were washed several times with water and centrifuged in order to eliminate polymers and secondary crystallizations from the matter solution. The water-dispersed powders were then sonicated for 1 min and dropped onto standard carbon-copper grids.

#### 2.3.5. Preliminary Studies of the Remineralization Potential

The preparation of the enamel samples was in accordance with the methodology described by Bonchev et al. [31].

The erupted third molars were used to create artificial enamel lesions. The radicular part of each tooth was removed, and the coronal part was separated in two halves (buccal and lingual). The buccal halves were polished with SiC paper disks with grit sizes of 320, 600, and 1200 (Shofu, Super-Snap Rainbow Technique Kit, Kyoto, Japan). The teeth samples were covered with two layers of acid-resistant nail varnish (Jerusalem, Israel), but a flat window with size of 4 × 4 mm was left uncovered. Then, the buccal part was separated into two symmetrical pieces. The demineralization was performed by using an aqueous solution of lactic acid (0.1 mM), NaH_2_PO_4_ (2.2 mM), CaCl_2_ (2.2 mM), and sodium fluoride (0.2 ppm) with a pH adjusted to 4.5. The solution was renewed every 24 h, and the enamel samples were immersed in it for 6 days, creating carious enamel lesions with a depth of <100 µm. 

The demineralized samples were rinsed with distilled water, sonicated for 5 min, and stored at 4 °C in distilled water prior to use. Following this preparation model, the de- and remineralization processes were compared with two halves from one and the same tooth.

The remineralization procedure was performed with freshly prepared suspensions of PCB/CaP and PSB/CaP hybrid materials synthesized in Series A. The powdered hybrid material was mixed with distilled water at a ratio of 0.1:2 g/mL for PCB/CaP and 0.1:1.25 g/mL for PSB/CaP to obtain thick suspensions. The specimens were covered either with the suspension of PCB/CaP or with suspensions PSB/CaP for 6 h, as described above. 

After the remineralization, each sample was carefully rinsed with distilled water and then stored in 6 mL of an artificial saliva solution [32] for 18 h. This procedure was repeated daily with a renewed artificial saliva solution for one week. 

#### 2.3.6. Micro-CT Scans

The de- and remineralized samples were scanned with the Bruker SkyScan 1272 X-ray scanner. The tube voltage and current were 90 kV and 111 µA, correspondingly. The voxel size was 2 µm. A tomographic reconstruction was performed with a dedicated software NRecon, version number 1.7.4.2. delivered by Bruker. A ring artefact and beam hardening corrections were applied. The beam hardening correction was adjusted by the tooth enamel. The 3D images before and after treatment were 3D-registered by DataViewer, also delivered by Bruker. A down sampling to a voxel size of 16 microns was necessary for this procedure. The region of interest containing the treatment windows was cut and registered again for better accuracy. The voxel size was 8 μm in the ROI registration. Absorption (density) profiles along a line 160 µm thick were taken in the 3D registration view simultaneously from the original ant-treated samples.

#### 2.3.7. Scanning Electron Microscopy (SEM) Analysis

The JEOL JSM 6390 apparatus was used for studying the morphology of the demineralized and remineralized tooth surfaces. The samples were gold-sputtered in vacuum, and SEM images were taken at several different magnifications.

#### 2.3.8. IR Reflection Micro-Spectroscopic Analysis

The non-treated, demineralized, and remineralized dental specimens were studied with the infrared microscope Hyperion 2000, Bruker Optik, Ettlingen, Germany in a spectral range of 600–4000 cm^−1^ with a 20× Schwarzschild objective in reflectance mode after accumulating 264 scans. Micro-infrared spectra were collected from five different areas, with mean size of 100 µm^2^ for each sample. 

#### 2.3.9. Raman Spectroscopy

Raman spectra of the remineralized samples were collected using the HR LabRam (Horiba, Jobin Yvon, Germany) spectrometer (600 grooves/mm grating) coupled with an Olympus optical microscope and a 50× objective in a range of 100–4000 cm^−1^. The 632.8 nm line of He-Ne laser was used for sample excitation. The Origin 9 software package was used for spectral evaluation.

## 3. Results

### 3.1. Effect of Betaine Functionality and Synthesis Route on the Precipitated Calcium Phosphate Phase Composition 

**Figure 1:**
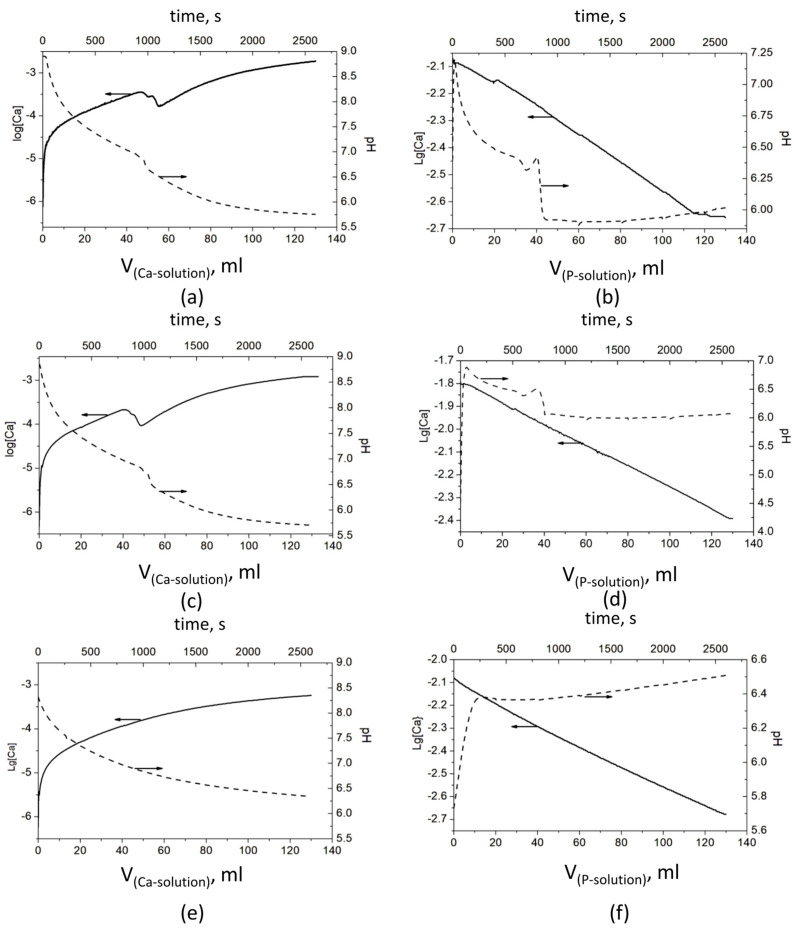
Evolution of pH and concentration of Ca^2+^ ions during the CaP precipitation process: Series A—(**a**) without a polymer, (**c**) with PSB, and (**e**) with PCB; Series B—(**b**) without a polymer, (**d**) with PSB, and (**f**) with PCB The arrows show which ordinate axis the data from the corresponding line belongs to.

The observed pH and Ca^2+^ ion concentration data in the polymer solutions, where CaP deposition took place, are presented in Figure 1. 

The results show significant differences in the behavior of the systems in both series—A and B. However, a similarity was observed in each of the series, between the polymer-free system and the system with PSB (Figure 1). In Series A, it is expressed in a bend in pH and especially in the concentration of Ca^2+^ ions between the added 45 and 65 mL Ca solution. The pH drops from 6.98 to 6.62 (Figure 1a) and from 6.86 to 6.48 (Figure 1c) and continues to decrease gradually to 5.7. At the same time, log[Ca] decreases in two steps from −3.45 to −3.55 and to −3.76 (Figure 1a) and from −3.67 to −3.77 and to −4.02 (Figure 1c). After that, log[Ca] increases smoothly to −2.73 and −2.90, respectively. In Series B, with the addition of the first portions of the P solution, the pH sharply increases by about 0.5 in the system without polymers (Figure 1b) and by almost two units in the system with PSB (Figure 1d). After that, the pH begins to gradually decrease until the addition of about 35 mL of the P solution, followed by a bend and a sharp decrease to about pH 6, at which value it remains relatively constant until the end of the precipitation process. In this series of experiments, the change in the concentration of the free Ca^2+^ ions is more difficult to notice due to the high concentration of the Ca^2+^ ions during the synthesis (the P solution is added to the Ca solution).

In the presence of PCB in Series A, (Figure 1e), the change of both parameters is smooth, with the pH falling from 8.9 to 6.5 and log[Ca] ions increasing from −6 to −3.27. By contrast, in Series B, the pH jumps from 5.7 to 6.4, with the first portion of the P solution, and slowly increases to 6.5 till the end of the experiment.

**Figure 2:**
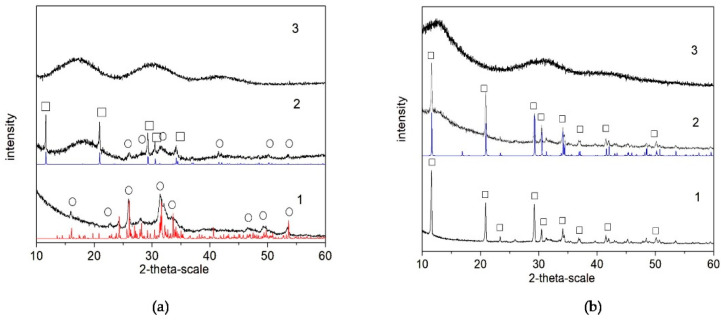
XRD studies of as-prepared biomaterials (after lyophilization) in the system: (**a**) Series A—(1) A-CaP, (2) A-PSB/CaP, and (3) A-PCB/CaP. (**b**) Series B—(1) B-CaP, (2) B-PSB/CaP, and (3) B-PCB/CaP. Red line (o)—powder diffraction data of OCP, ICSD Collection Code 65347; blue line (□)—powder diffraction data of DCPD, ICSD Collection Code 16132.

**Figure 3:**
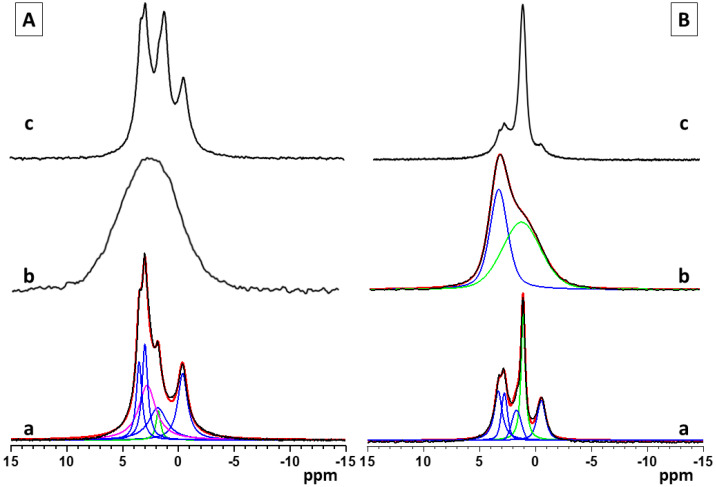
Single pulse ^31^P NMR spectra of pure and hybrid materials from Series A (**A**)—(**a**) pure A-CaP, (**b**) A-CaP/PCB, and (**c**) A-CaP/PSB—and Series B (**B**): (**a**) pure B-CaP, (**b**) B-CaP/PCB, and (**c**) B-CaP/PSB. The spectra of the pure A-CaP, pure B-CaP, and B-CaP/PCB samples were deconvoluted to identify the different components present in the mixture. Experimental spectra are presented with black lines, while the simulated spectra are given with red lines, and the individual contributions of the different phases obtained by the deconvolution of the spectral patterns are given with colored lines (OCP—blue, ACP/Hap—magenta, DCPD and acidic amorphous phase—green).

The results of the XRD analysis (Figure 2) of the final products (after lyophilization) show that, in the polymer-free systems, the calcium phosphate phase is Ca_8_(HPO_4_)_2_(PO_4_)_4_.5H_2_O (OCP, octacalcium phosphate) in Series A (Figure 2(a-1)) but CaHPO_4_.2H_2_O (DCPD, dicalcium phosphate dihydrate) in Series B (Figure 2(b-1)). In the presence of PSB, a mixture of OCP and CaHPO_4_.2H_2_O (DCPD, brushite) was obtained in Series A (Figure 2(a-2)), but again only DCPD was obtained in Series B (Figure 2(b-2)). In the presence of PCB, the phase remains XRD-amorphous in both series (Figure 2(a-3,b-3)).

The single pulse ^31^P and ^1^H→^31^P CP-MAS solid state NMR spectra provide a more detailed insight into the structural characteristics and chemical composition of the calcium phosphate phases formed in the studied materials, as a function of the sample preparation procedure, synthesis conditions, and the polymer functional groups. The single pulse ^31^P spectra give information about all types of CaP phases, while in the ^1^H→^31^P CP-MAS, the resonances of the P atoms with closely spaced protons, such as hydrogen phosphate species, are specifically enhanced due to the transfer of magnetization to the P atom from the neighboring protons. The relative areas of the characteristic resonances in the single pulse ^31^P spectra give quantitative information about the relative molar fractions of the different CaP phases. The standard ^1^H→^31^P CP-MAS spectra, however, cannot be interpreted quantitatively since the effectiveness of cross-polarization transfers depends on the P---H internuclear distance, the relaxation rates, and the local dynamics of the structural fragments, which vary from one chemical environment to another.

Figure 3(Aa–c) shows the single pulse ^31^P NMR spectra of pure calcium phosphate and the hybrid CaP materials synthesized with the two polymers, PCB and PSB, in Series A. The NMR spectrum of the pure A-CaP (Figure 3(Aa)) indicates the formation of the mixture of several phases. The deconvolution of the spectrum shows the presence of OCP as a main component (signals at −0.4, 2.9, 3.2, and around 4.5 ppm) as well as some amounts of ACP and/or poorly crystalline HA (resonance at around 2.9 ppm) and of DCPD (1.3 ppm). The addition of PCB (sample A-Cap/PCB) results in the formation of an amorphous phase, represented by a broad resonance centered at 2.6 ppm, characteristic for apatite phosphates (Figure 3(Ab)). The ^31^P spectral pattern of the hybrid material, A-CaP/PSB synthesized in the presence of PSB (Figure 3(Ac)), is very similar to those of the pure A-CaP obtained by the same synthesis procedure; however, in this case, the intensity of the resonance at 1.3 ppm is higher, indicating the formation of a higher amount of DCPD as compared to the pure CaP material. The presence of OCP and DCPD in the pure A-CaP and in the hybrid material, A-Cap/PSB, was further confirmed by the ^1^H→^31^P CP-MAS spectra (Supplementary Materials, Figure S3a,b). In the ^1^H→^31^P CP-MAS spectra, the resonances of the P atoms of the hydrogen phosphate groups from OCP at −0.4 ppm and from the DCPD at 1.3 ppm were specifically enhanced due to the transfer of magnetization to the P atom from the neighboring acidic protons of the hydrogen phosphate groups. 

The ^31^P spectra of pure calcium phosphate (B-CaP) and the hybrid materials synthesized in the presence of PCB (B-CaP/PCB) and PSB (B-CaP/PSB) from Series B are presented in Figure 3(Ba–c). Under the used synthesis conditions, the pure B-CaP contains a mixture of OCP and nano-crystalline DCPD, represented by the relatively sharp resonance at 1.3 ppm (Figure 3(Ba)). The ^31^P spectrum of the hybrid material, B-CaP/PCB, shows a broad non-symmetrical resonance, indicating the formation of at least two different phases (Figure 3(Bb)). Two main peaks could be identified after the deconvolution of the spectrum: a resonance centered at around 2.9 ppm, characteristic of poorly crystalline HA and/or ACP, which is overlapped by a broad second resonance centered at around 1.7 ppm, indicating the formation of a non-stoichiometric amorphous phase of acidic phosphates.

The relative molar ratios of the two resonances were 25:75, respectively. The presence of the acidic amorphous phase was further confirmed by the ^1^H→^31^P CP-MAS spectra, where a broad symmetric resonance centered at around 1.7 ppm was observed at all mixing times (Supplementary Materials, Figure S4a). The different intensities of the two resonances in the single pulse and the ^1^H→^31^P C MAS spectra could be explained with the specific enhancement of the signal at 1.7 ppm due to the transfer of magnetization to the P atom from the neighboring protons in the -HPO_4_ moiety. The enhancement of the resonance at 2.9 ppm is less pronounced at short mixing times and is clearly visible only at longer mixing times (Supplementary Materials, Figure S4a,b). In the HA structure, the distance between the P atoms and neighboring protons is larger since the protons are not a part of the phosphate groups and, therefore, the transfer of magnetization is less efficient as compared to the acidic phosphate phases. The single pulse ^31^P (Figure 3(Bc)) and the ^1^H→^31^P CP-MAS spectra (Figure S4c,d) of the hybrid material, B-CaP/PSB, display similar spectral patterns as observed for the pure B-CaP phase; however, in the presence of PSB, a higher amount of DCPD was detected.

### 3.2. Characterization and Actions of a Newly Synthesized Hybrid Material

Two hybrid biomaterials were chosen for compositional and morphological characterization and preliminary studies of their remineralization potential, namely, the ones obtained in Series A, due to the absence of the non-stoichiometric amorphous phase of acidic phosphates in A-PCB/CaP and lower amounts of DCPD in A-PSB/CaP than in B-PSB/CaP. The presence of acid phosphates is not characteristic of normal calcification processes.

#### 3.2.1. Composition and Morphology 

TEM with EDS and DTA-TG-MASS analyses were performed to characterize the morphology and the composition of the new hybrid materials.

**Figure 4:**
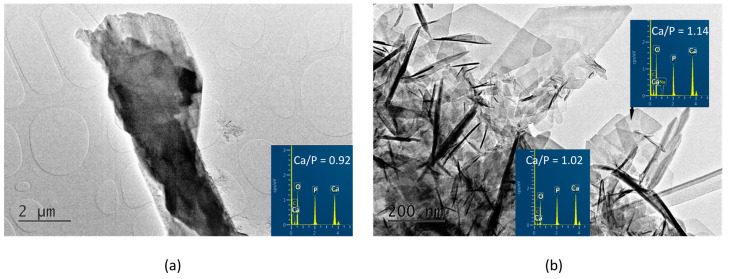
TEM images and EDS analyses of the A-PSB/CaP (**a**) column-like crystal with Ca/P ratio of 0.92; (**b**) needle-shaped crystals with Ca/P ratio of 1.02 and plate-like crystals with Ca/P ratio of 1.14.

In the A-PSB/CaP material, three types of particles are observed that differ in shape, size, and molar Ca/P ratio, namely: (i) micro-sized single plate-like particles (Figure 4a) with Ca/P = 0.92; (ii) needle shaped particles with Ca/P = 1.02; and (iii) sheet-like particles with Ca/P = 1.14 (Figure 4b). 

**Figure 5:**
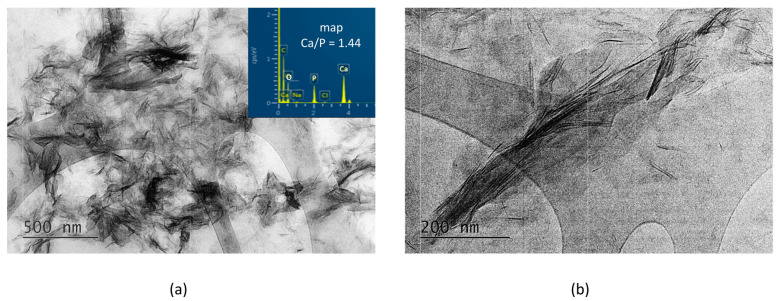
TEM images at different magnifications and EDS analysis of the particles of A-PCB/CaP. (**a**) magnification 10 kx; (**b**) magnification 30 kx.

In contrast, in the A-PCB/CaP materials, only one type of fine needle-like particle with Ca/P = 1.44 is visible in the TEM images (Figure 5). Unlike the randomly scattered particles obtained in the presence of PSB (Figure 4b), these are arranged in bundles of uniformly oriented particles (Figure 5b).

**Figure 6:**
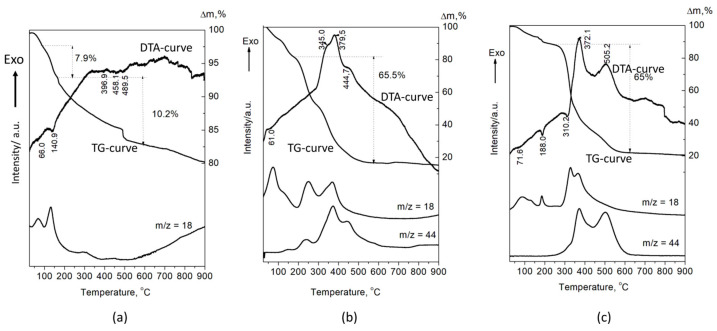
DTA-TG-MASS analysis of the (**a**) A-CaP, (**b**) A-PSB/CaP, and (**c**) A-PCB/CaP.

The DTA-TG-MASS analysis of the A-CaP (Figure 6a) shows the decomposition of OCP (T_max_ = 140.9 °C and weight loss of 7.9 mas%), according to the following reaction: 

Ca_8_(HPO_4_)_2_(PO_4_)_4_.5H_2_O → 0.5Ca_10_(PO_4_)_6_(OH)_2_ + 3CaHPO_4_ + 4H_2_O [33]. 

The next endothermic peaks, accompanied with a weight loss of 10.2%, are due to the decomposition of CaHPO_4_. The DTA curves of A-PSB/CaP and A-PCB/Ca-P are characterized with strong exothermic effects in a temperature range of 200–600 °C, which can be attributed to the burning of the polymers (Figure 6b,c). They are accompanied with a weight loss of 65 and 65.5%, respectively, and a release of CO_2_ and H_2_O. The effects of the decomposition of the calcium phosphate compounds in HMs are overlapped by the effects of polymer combustion. This makes it difficult to accurately determine the amount of polymer in the two materials. However, it can be assumed that it is 50–55 mas %, determined by the differences in weight loss of A-PSB/CaP or A-PCB/CaP with A-CaP in the temperature region 200–600 °C. 

#### 3.2.2. Preliminary Studies on the Remineralization Potential of the Selected Hybrid Materials

The mineral loss after the demineralization procedure and the mineral uptake after the remineralization procedure of the enamel were monitored non-destructively using the micro-CT analysis.

**Figure 7:**
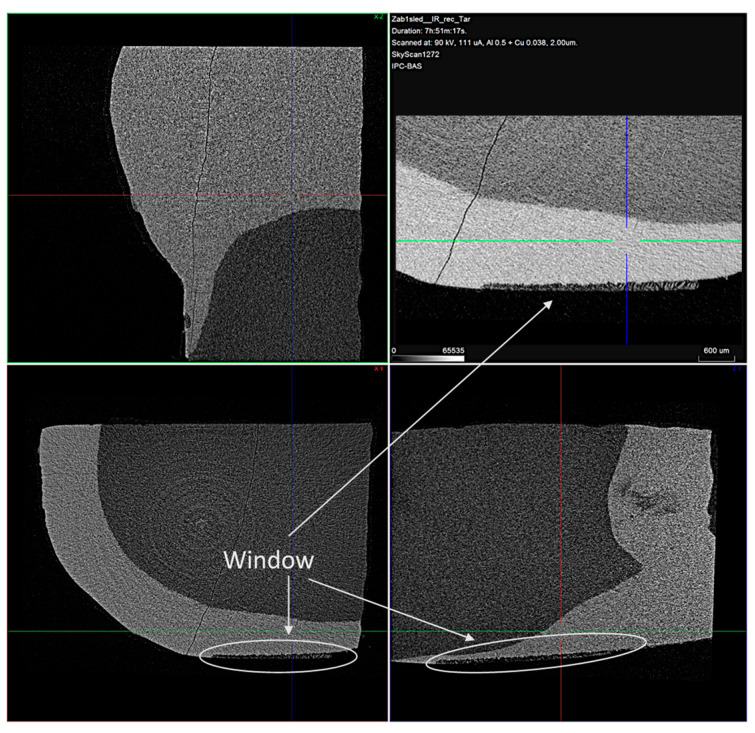
Typical cross-sectional view of the samples with the treatment window enlarged. Voxel size is 2 μm.

The typical 2D micro-CT images of the samples are shown in Figure 7. 

In the cross-sectional images, a distinct faint shadow in the enamel (arrows) is observed, which is due to the formed enamel lesion. From the measurements made, it was found that the resulting lesions were located in the superficial enamel layer. The initial lesion depth of the samples was in a range of 30–90 µm.

**Figure 8:**
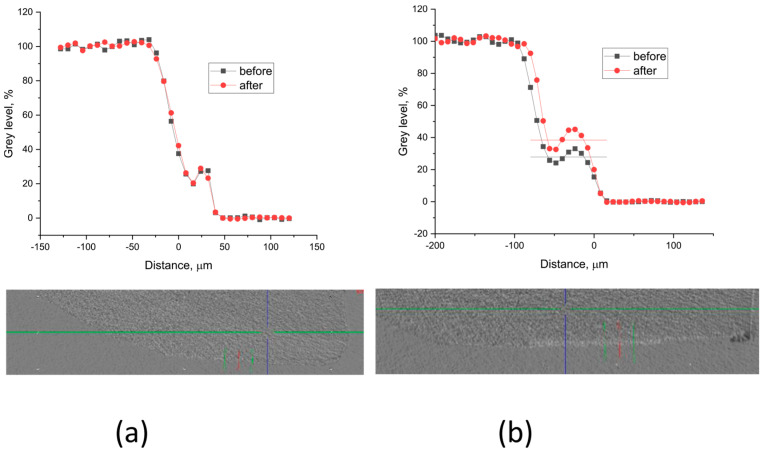
Typical gray level profiles of the non-treated (black line) and treated (red line) specimens with (**a**) A-PSB/CaP—density increase 0%, thickness ~ 40 µm; (**b**) A-PCB/CaP—density increase 14%, thickness ~ 56 µm.

**Figure 9:**
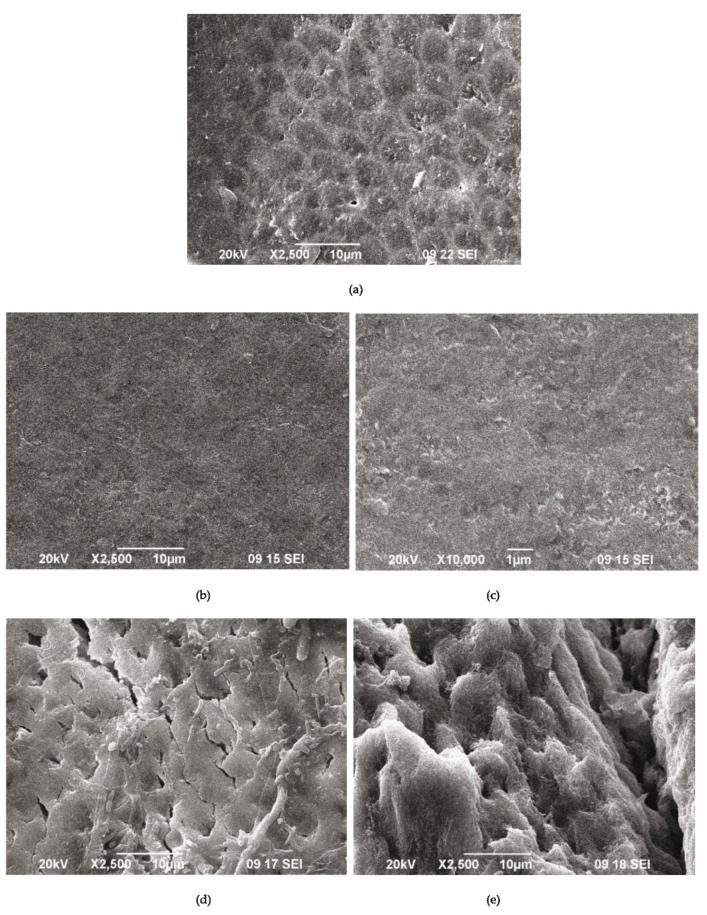
SEM images of demineralized enamel (**a**) after the remineralization procedure with A-PSB/CaP (**b**,**c**) and after the remineralization procedure with A-PCB/CaP (**d**,**e**).

Grey level profiles of the treated and non-treated samples are shown in Figure 8 and Figure 9. The profiles are scaled so that the background is 0, but the enamel gray level is 100. This allows for the etched sample area in the window to appear as a step. The density (the X-ray absorption ability) increase after treatment is estimated as part of the etched density. 

The results show that in the samples treated with A-PSB/CaP, no increase in density was identified (Figure 8a) in the lesions in contrast to those treated with A-PCB/CaP (Figure 8b). 

The SEM images of the demineralized enamel (Figure 9a) and after the remineralization procedures (Figure 9b–f) show different behaviors of both materials. 

The SEM images of the demineralized enamel showed a dissolution predominantly of the central part of the enamel rods and a widening of the inter-rod zones (Figure 9a). The porous structure of the demineralized enamel changed into a dense flat surface as a result of using A-PSB/CaP (Figure 9b,c). The samples treated with A-PCB/CaP showed a heterogeneous structure due to crystal accumulation and the formation of scattered granular deposits. The newly formed layer is similar to the structure of the underlying enamel (Figure 9d). 

A porous net-like substance with a width of the pores up to 2 μm is observed on the surface of the substrates treated with A-PCB/CaP. We hypothesize that the porous structure allows for the penetration of the hydrogel suspension, thus allowing for the deposition of the needle-like crystals of ACP in the demineralized enamel rods (Figure 9e).

**Figure 10:**
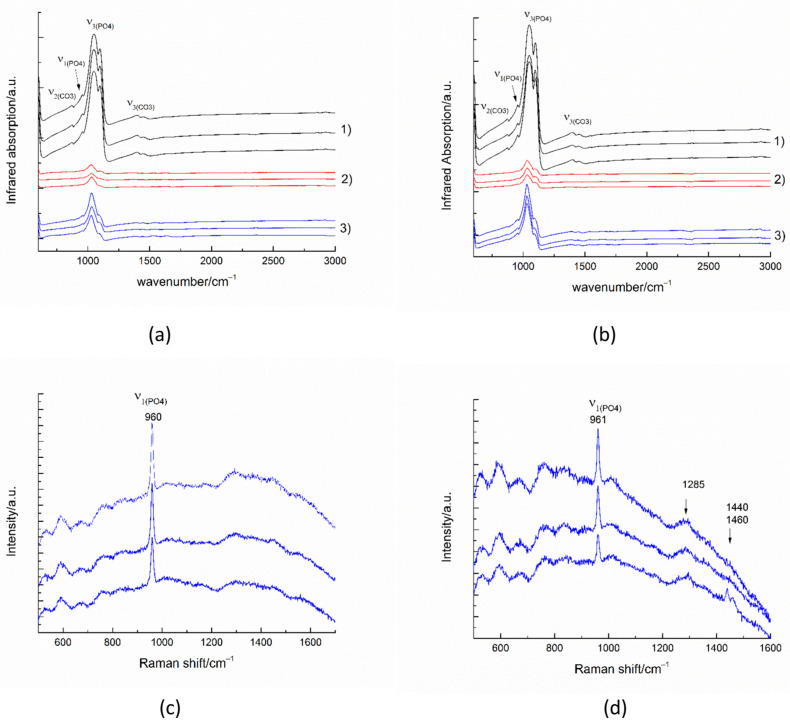
IR spectra (**a**,**b**) and Raman spectra (**c**,**d**) of substrates after the remineralization procedure with A-PCB/CaP (**a**,**c**) and after the remineralization procedure with A-PSB/CaP (**b**,**d**). (1) untreated substrate, (2) demineralized substrate, and (3) remineralized substrate.

The infrared spectra collected in reflectance mode from 100 × 100 micrometer areas of untreated enamel (Figure 10(a-1,b-1)) show the strong characteristic peaks of antisymmetric *ν*_3_(PO_4_) stretching between 1027–1100 cm^−1^. The weak peak near 957 cm^−1^ results from the symmetric *ν*_1_(PO_4_) stretching in the apatite. The peaks near 870 cm^−1^ and between 1410–1540 cm^−1^ are generated by the out-of-plane *ν*_2_ vibration of CO_3_ and the antisymmetric *ν*_3_(CO_3_) stretching impurities in the apatite, respectively [34].

The infrared spectra of the remineralized surfaces (Figure 10(a-3,b-3)) show a significant increase in intensity compared to the demineralized surfaces. The formation of a smoother surface was also confirmed by the SEM images. 

The most intense enamel peaks are found in the range of (PO_4_) stretching. The Raman spectra of the remineralized surfaces (Figure 10c,d) show the most intense enamel peak, generated by the symmetric ν_1_(PO_4_) stretching in the apatite and some traces of the polymer matrix. The peak around 1455 cm^−1^ may be due to CH_2_ bending.

## 4. Discussion

### 4.1. Effects of Betaine Functionality and Synthesis Routes on the Precipitated CaP Phases

The effects of the betaine functionalities and the synthesis route on the type of the precipitated calcium phosphates were evaluated during the precipitation process. The varied way of solution mixing has an effect on the initial pH and the microenvironment for CaP’s precipitation during the synthesis and hence on the type of pre-nucleation clusters, according to the non-classical theory of CaP crystallization [35]. 

In the case of Series A, where the Ca solution was added to the P solution, HPO_4_^2−^ ions dominate at the beginning and the formation of Ca(HPO_4_)_3_^4−^ pre-nucleation clusters is expected to prevail over other ones [36]. The starting pH is high (a pH of 8.9, Figure 1a,c,e) which results in the formation of amorphous calcium phosphate with the common formula Ca_x_(HPO_4_)_y_(PO_4_)_z_ and Ca/P = 1.1–1.6 [37].

In the case of Series B (the P solution was added to the Ca solution), Ca^2+^ ions prevail in the initial solution. The pH was 4.5 for the system with PSB (Figure 1d), 5.7 for the system with PCB (Figure 1f), and 6.3 for the polymer-free system (Figure 1b). Thus, it was more probable that CaHPO_4_^0^ pre-nucleation clusters, which are structural units of DCPD, would be dominant [38]. Further, the composition of the pre-nucleation clusters, and the solid phase formed by them, changed depending on the conditions in the systems. 

The different course of the changes in pH and in the concentration of the free Ca^2+^ ions during precipitation in the presence of PSB or PCB in both series (Figure 1) is proof of the influence of the distinct nature of the negatively charged functional group which results in different dipole moments of the formed zwitterionic (betaine) structure. In the case of the polymer-free system or in the presence of PSB, which has a sulfo group that is a stronger acidic group as compared to the carboxylic group, the pH changes to a greater extent (Figure 1a–d). The step change in the concentration of the free Ca^2+^ ions (Figure 1a–d) is an indication of the formation of different pre-nucleation clusters during the synthesis. 

The carboxylic group of PCB enters in acid–base interactions, acting as a buffer, which explains the gradual and smaller pH changes observed for the PCB-controlled CaP precipitation, accompanied by a smooth change in the free Ca^2+^ concentration (Figure 1e,f). Also, the polymer macromolecules of PCB interact more actively with the formed pre-nucleation clusters than those of PSB. According to Gebauer [35], the interactions can be either adsorption on the surface or complexation with the Ca^2+^ ions. Thus, the formation of new clusters is hindered, and the formation of amorphous intermediate phases is promoted. In the PSB system, such interactions are suppressed. The dipole moment of the PSB zwitterionic structure is weaker than the PCB one and determines weaker interactions with the surrounding ions and water molecules. Moreover, the smaller size of the CaHPO_4_^0^ clusters than the Ca(HPO_4_)_3_^4−^ ones inhibits polymer adsorption. In addition, the more acidic character of the sulfo group than the carboxy one determines the lower pH during the synthesis and dominance of the acidic solid phases in the precipitates in the system with PSB (Figure 2 and Figure 3).

### 4.2. Characterization and Actions of a Newly Synthesized Hybrid Material

Two newly synthesized hybrid materials were chosen to be characterized and for preliminary testing of their remineralization potential, namely, A-PCB/CaP and A-PSB/CaP, due to the absence of an acidic calcium phosphate phase in A-CaP/PCB and the smaller amount of DCPD in A-CaP/PSB (Figure 3). The Acidic phases are not characteristic of normal calcifications in the human body, although DCPD and OCP are considered precursors for obtaining HA, along with ACP [39,40]. The CaP-to-polymer ratio in both materials is 1:1 (Figure 6b,c). The mineral phase, A-PCB/CaP, contains ACP (Figure 2 and Figure 3) with thin acicular, uniformly oriented particles arranged in bundles (Figure 5). A-PSB/CaP contains a mixture of OCP and DCPD (Figure 2 and Figure 3) with larger-sized and differently shaped particles (Figure 4).

Remineralization is the process by which the enamel crystals must regain their size, shape, and strength. Non-fluoride approaches imply the involvement of calcium and phosphate ions from a source external to the tooth to promote ion deposition on the enamel crystals [41]. Such a source is saliva, but the amount of calcium and phosphate ions in saliva is not sufficient for complete remineralization [42]; thus, pre-formed crystals of nano HA or other types of calcium phosphates were used, along with bioactive organic substances, for targeting mineral deposits [43]. 

The use of a suspension of the hybrid materials, prepared in this study, was intended to induce the penetration of the polymer hydrogel through the thinned enamel layer, which would aid both the introduction and orientation of the preliminarily obtained CaP particles, and the activation of the nucleation centers in the residual enamel. Thus, during the next cycle of immersion in artificial saliva, the processes of crystallization and re-crystallization in HA promoted the faster recovery of the enamel to be stimulated. This strategy needs to be carefully balanced to prevent the clogging of the tubules and the uptake of mineral ions into the lesion.

The comparative study of the remineralization potential of the two materials gives an advantage to A-PCB/CaP. Under the conditions of the experiment, it leads to the creation of a newly formed crystal layer similar to that of the underlying enamel (Figure 9e) and an increase in density in the lesion (Figure 8b). No extraneous mineral phases were found after the remineralization procedure except hydroxyapatite forming the tooth enamel (Figure 10). 

The formation of more than one phase during the CaP synthesis within the presence of PSB increases the prospects of the obtained material (A-PSB/CaP) to be successful as a remineralization system because it contains two phases, namely, OCP and DCPD, which are both HA precursors. Moreover, due to their higher solubility, as compared to HA, they are both able to act as ion-releasing systems. According to us, the different solubility of OCP and DCPD [37] would lead to a continuous, gradual release of Ca^2+^ and PO_4_^3−^ ions for enhancing the enamel remineralization. The advantage of A-PCB/CaP’s performance could be sought rather in the ACP phase, where it prevails, as ACP is known to be unstable, and in an aqueous media, it is easily transformed into stable crystalline phases, such as octacalcium phosphate or apatite, due to the growing of microcrystalline ones [44]. Thus, the type of the CaP phase is the dominant factor defining the success of hybrid materials as remineralization systems.

Mineral phase deposition rather than enamel remineralization was observed as a result of our preliminary investigations. Although further in-depth studies are needed, including a variation of the suspension density and contact time, A-PCB/CaP shows promising properties for a material applicable in dentistry enamel remineralization.

The information gathered from this study can be used to plan future studies to test materials, but its findings cannot be used to guide clinical judgments. The effectiveness of these materials for remineralizing teeth needs to be further investigated, as in a laboratory environment, in clinical conditions too.

## 5. Conclusions

The effects of betaine zwitterionic functionality and the synthesis route on the biomimetic precipitation of CaP phases were investigated in this study by the monitoring of pH and Ca^2+^ ion concentration during the synthesis along with X-ray powder diffraction and solid-state NMR analyses. Further, preliminary studies on the remineralization potential of the selected new synthesized hybrid materials were carried out, and their effects were characterized by MictoCT, SEM, and IR analyses. 

Both polymers used by us, PCB and PSB, have positively charged ammonium groups and negatively charged acidic, carboxy, or sulfo groups covalently bonded to their side chains, respectively. The different chemical behaviors of these groups in the solution determine their different interactions with the surrounding ions and with the pre-nucleation clusters formed during the synthesis. Thus, ACP was obtained to precipitate in the PCB-controlled system, while OCP and DCPD in different ratios were obtained in the PSB or polymer-free systems. The findings of our study demonstrate the significance of organic component functional groups in the biomineralization process.

The preliminary comparative study of the remineralization potential of the two as-prepared hybrid materials gives preference to that consisting of amorphous calcium phosphate and PCB. Under the conditions of the experiment, it leads to the creation of a newly formed crystal layer similar to that of the underlying enamel and an increase in density in the lesion.

Our research is a basis for building a future strategy for a more in-depth study of the remineralization potential of the obtained materials.

## Data Availability

The data presented in this study are available in this article and in the Supplementary Materials.

## Supplementary Materials

The following supporting information can be downloaded at: https://www.mdpi.com/1996-1944/16/20/6640/s1, Scheme S1. RAFT polymerization to obtain PCB, Scheme S2. RAFT polymerization to obtain PSB, Figure S1. ^1^H spectrum of PCB in D2O, Figure S2. ^1^H spectrum of PSB in D2O, Figure S3. ^1^H→^31^P CP-MAS spectra of pure A-CaP and hybrid A-CaP/PSB materials from Series A. Figure S4. ^1^H→^31^P CP-MAS spectra of materials from Series B: pure B-CaP, hybrid B-CaP/PSB material, and hybrid B-CaP/PCB material measured at mixing times of 100 µs and 4 ms; The spectra are discussed in detail in the main text of the paper.

## References

[1] Sharma V., Srinivasan A., Nikolajeff F., Kumar S. (2021). Biomineralization process in hard tissues: The interaction complexity within protein and inorganic counterparts. Acta Biomater..

[2] Bartlett J.D., Smith C.E., Hu Y., Ikeda A., Strauss M., Liang T., Hsu Y.-H., Trout A.H., McComb D.W., Freeman R.C. (2021). MMP20-generated amelogenin cleavage products prevent formation of fan-shaped enamel malformations. Sci. Rep..

[3] Shaw W.J., Tarasevich B.J., Buchko G.W., Arachchige R.M.J., Burton S.D. (2020). Controls of nature: Secondary, tertiary, and quaternary structure of the enamel protein amelogenin in solution and on hydroxyapatite. J. Struct. Biol..

[4] Tao J., Hanson E., Dohnalkova A.C., Buchko G.W., Jin B., Shaw W.J., Tarasevich B.J. (2022). Changes in the Cterminal, N-terminal, and histidine regions of amelogenin reveal the role of oligomer quaternary structure on adsorption and hydroxyapatite mineralization. Front. Physiol..

[5] Beniash E., Metzler R.A., Lam R.S., Gilbert P.U. (2009). Transient amorphous calcium phosphate in forming enamel. J. Struct. Biol..

[6] Hamzehlou S., Aboudzadeh M.A. (2021). Special Issue on “Multifunctional Hybrid Materials Based on Polymers: Design and Performance. Processes.

[7] Zhao M., Geng Y., Fan S., Yao X., Zhu M., Zhang Y. (2021). 3D-printed strong hybrid materials with low shrinkage for dental restoration. Compos. Sci. Technol..

[8] Prosheva M., Aboudzadeh M.A., Leal G.-P., Gilev J.B., Tomovska R. (2019). High-Performance UV Protective Waterborne Polymer Coatings Based on Hybrid Graphene/Carbon Nanotube Radicals Scavenging Filler. Part. Part. Syst. Charact..

[9] Hench L.L., Jones J.R. (2015). Bioactive Glasses: Frontiers and Challenges. Front. Bioeng. Biotechnol..

[10] Santos-Coquillat A., Martínez-Campos E., Sánchez H.M., Moreno L., Arrabal R., Mohedano M., Gallardo A., Rodríguez-Hernández J. (2021). Matykina, Hybrid functionalized coatings on Metallic Biomaterials for Tissue Engineering. Surf. Coat. Technol..

[11] Xu J., Shi H., Luo J., Yao H., Wang P., Li Z., Wei J. (2022). Advanced materials for enamel remineralization. Front. Bioeng. Biotechnol..

[12] Combes C., Rey C. (2010). Amorphous calcium phosphates: Synthesis, properties and uses in biomaterials. Acta Biomater..

[13] Salama A. (2019). Cellulose/calcium phosphate hybrids: New materials for biomedical and environmental applications. Int. J. Biol. Macromol..

[14] Wang W., Zhang B., Zhao L., Li M., Han Y., Wang L., Zhang Z., Li J., Zhou C., Liu L. (2021). Fabrication and Properties of PLA/Nano-HA Composite Scaffolds with Balanced Mechanical Properties and Biological Functions for Bone Tissue Engineering Application. Nanotechnol. Rev..

[15] Pupilli F., Ruffini A., Dapporto M., Tavoni M., Tampieri A., Sprio S. (2022). Design Strategies and Biomimetic Approaches for Calcium Phosphate Scaffolds in Bone Tissue Regeneration. Biomimetics.

[16] Hoshi M., Taira M., Sawada T., Hachinohe Y., Hatakeyama W., Takafuji K., Tekemoto S., Kondo H. (2022). Preparation of Collagen/Hydroxyapatite Composites Using the Alternate Immersion Method and Evaluation of the Cranial Bone-Forming Capability of Composites Complexed with Acidic Gelatin and b-FGF. Materials.

[17] Bartmanski M., Rosciszewska M., Wekwejt M., Ronowska A., Nadolska-Dawidowska M., Mielewczyk-Gryn A. (2022). Properties of New Composite Materials Based on Hydroxyapatite Ceramic and Cross-Linked Gelatin for Biomedical Applications. Int. J. Mol. Sci..

[18] Örlygsson G., Laxdal E.H., Kárason S., Dagbjartsson A., Gunnarsson E., Ng C.H., Einarsson J.M., Gíslason J., Jónsson H. (2022). Mineralization in a Critical Size Bone-Gap in Sheep Tibia Improved by a Chitosan-Calcium Phosphate-Based Composite as Compared to Predicate Device. Materials.

[19] Zagho M.M., Hussein E.A., Elzatahry Á.A. (2018). Recent overviews in functional polymer composites for biomedical applications. Polymers.

[20] Furko M., Balázsi K., Balázsi C. (2023). Calcium Phosphate Loaded Biopolymer Composites—A Comprehensive Review on the Most Recent Progress and Promising Trends. Coatings.

[21] Jee S.S., Thula T.T., Gower L.B. (2010). Development of bone-like composites via the polymer-induced liquid-precursor (PILP) process. Part 1: Influence of polymer molecular weight. Acta Biomater..

[22] Katti K.S., Ambre A.H., Peterka N., Katti D.R. (2010). Use of unnatural amino acids for design of novel organomodified clays as components of nanocomposite biomaterials. Philos. Trans. R. Soc. A Math. Phys. Eng. Sci..

[23] Palazzo B., Walsh D., Iafisco M., Foresti E., Bertinetti L., Martra G., Bianchi C.L., Cappelletti G., Roveri N. (2009). Amino acid synergetic effect on structure, morphology and surface properties of biomimetic apatite nanocrystals. Acta Biomater..

[24] Goloshchapov D., Kashkarov V., Nikitkov K., Seredin P. (2021). Investigation of the Effect of Nanocrystalline Calcium Carbonate-Substituted Hydroxyapatite and L-Lysine and L-Arginine Surface Interactions on the Molecular Properties of Dental Biomimetic Composites. Biomimetics.

[25] Liu P., Song J. (2013). Sulfobetaine as a zwitterionic mediator for 3D hydroxyapatite mineralization. Biomaterials.

[26] Liu P., Emmons E., Song J. (2014). A comparative study of zwitterionic ligands-mediated mineralization and the potential of mineralized zwitterionic matrices for bone tissue engineering. J. Mater. Chem. B Mater. Biol. Med..

[27] Słota D., Florkiewicz W., Piętak K., Pluta K., Sadlik J., Miernik K., Sobczak-Kupiec A. (2022). Preparation of PVP and betaine biomaterials enriched with hydroxyapatite and its evaluation as a drug carrier for controlled release of clindamycin. Ceram. Int..

[28] Mangal U., Kwon J.-S., Choi S.-H. (2020). Bio-Interactive Zwitterionic Dental Biomaterials for Improving Biofilm Resistance: Characteristics and Applications. Int. J. Mol. Sci..

[29] Ruseva K., Ivanova K., Todorova K., Vladov I., Nanev V., Tzanov T., Hinojosa-Caballero D., Argirova M., Vassileva E. (2020). Antibiofilm poly(carboxybetaine methacrylate) hydrogels for chronic wounds dressings. Eur. Polym. J..

[30] Massiot D., Fayon F., Capron M., King I., Le Celve S., Alonson B., Durand J.O., Bujoli B., Gan Z.H., Hoatson G. (2002). Modelling one- and two-dimensional solid-state NMR spectra. Magn. Reson. Chem..

[31] Bonchev A., Simeonov M., Shestakova P., Vasileva R., Titorenkova R., Apostolov A., Dyulgerova E., Vassileva E. (2022). Bioinspired Remineralization of Artificial Caries Lesions Using PDMAEMA/Carbomer/Calcium Phosphates Hybrid Microgels. Gels.

[32] Klimek J., Hellwig E., Ahrens G. (1982). Fluoride taken up by plaque, by the underlying enamel and by clean enamel from three fluoride compounds in vitro. Caries Res..

[33] Yokoi T., Goto T., Kato T., Takahashi S., Nakamura J., Sekino T., Ohtsuki C., Kawashita M. (2020). Hydroxyapatite Formation from Octacalcium Phosphate and Its Related Compounds: A Discussion of the Transformation Mechanism. Bull. Chem. Soc. Jpn..

[34] Jegova G., Titorenkova R., Rashkova M., Mihailova B. (2013). Raman and IR reflection micro-spectroscopic study of Er: YAG laser treated permanent and deciduous human teeth. J. Raman Spectrosc..

[35] Gebauer D. (2018). How Can Additives Control the Early Stages of Mineralisation?. Minerals.

[36] Habraken W., Tao J., Brylka L., Friedrich H., Bertinetti L., Schenk A.S., Verch A., Dmitrovic V., Bomans P.H.H., Frederik P.M. (2013). Ion-association complexes unite classical and non-classical theories for the biomimetic nucleation of calcium phosphate. Nat. Commun..

[37] Dorozhkin S.V. (2010). Amorphous calcium (ortho)phosphates. Acta Biomater..

[38] Rabadjieva D., Sezanova K., Gergulova R., Titorenkova R., Tepavitcharova S. (2020). Precipitation and phase transformation of dicalcium phosphate dihydrate in electrolyte solutions of simulated body fluids: Thermodynamic modeling and kinetic studies. J. Biomed. Mater. Res. Part A.

[39] Lakrat M., Jodati H., Mejdoubi E.M., Evis Z. (2023). Synthesis and characterization of pure and Mg, Cu, Ag, and Sr doped calcium-deficient hydroxyapatite from brushite as precursor using the dissolution-precipitation method. Powder Technol..

[40] Rahmani F., Larbi Bouamrane O., Ben Bouabdallah A., Atanase L.I., Hellal A., Apintiliesei A.N. (2023). Biomimetic Hydroxyapatite Crystals Growth on Phosphorylated Chitosan Films by In Vitro Mineralization Used as Dental Substitute Materials. Polymers.

[41] Cochrane N.J., Cai F., Huq N.L., Burrow M.F., Reynolds E.C. (2010). New approaches to enhanced remineralization of tooth enamel. J. Dent. Res..

[42] Reynolds E.C. (2008). Calcium phosphate-based remineralization systems: Scientific evidence?. Aust. Dent. J..

[43] Carey C.M. (2023). Remineralization of Early Enamel Lesions with Apatite-Forming Salt. Dent. J..

[44] Zhao J., Liu Y., Sun W.B., Zhang H. (2011). Amorphous calcium phosphate and its application in dentistry. Chem. Cent. J..

