# Supplementary material for: Polycarboxy/Sulfo Betaine—Calcium Phosphate Hybrid Materials with a Remineralization Potential
Source: Materials (Basel). 2023 Oct 11;16(20):6640. doi: 10.3390/ma16206640 (PMC10608424; doi:10.3390/ma16206640)
Supplement: Supplementary file 1 [file materials-16-06640-s001.zip › materials-2642392-supplementary.pdf]

SUPPLEMENTARY MATERIAL

List of Figures in Supplementary Material:

Scheme S1: RAFT polymerization to obtain PCB

Scheme S2: RAFT polymerization to obtain PSB

Figure S1: ^{1}H spectrum of PCB in D_2O

Figure S2: ^{1}H spectrum of PSB in D_2O

Figure S3. $^{1}H \rightarrow ^{31}P$ CP-MAS spectra of pure A-CaP and hybrid A-CaP/PSB materials from series A.

Figure S4. $^{1}H \rightarrow ^{31}P$ CP-MAS spectra of materials from series B: pure B-CaP, hybrid B-CaP/PSB material; and hybrid B-CaP/PCB material measured at mixing times of 100 µs and 4 ms; The spectra are discussed in details in the main text of the paper.

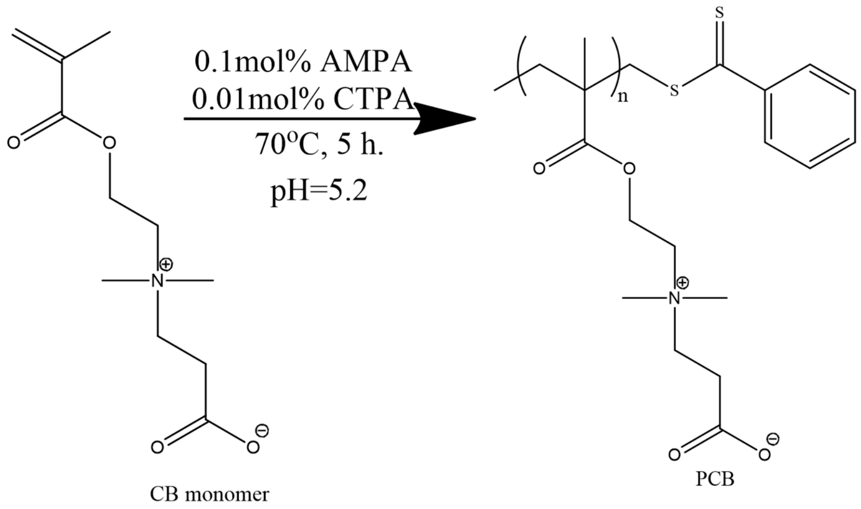

Scheme S1. RAFT polymerization to obtain PCB

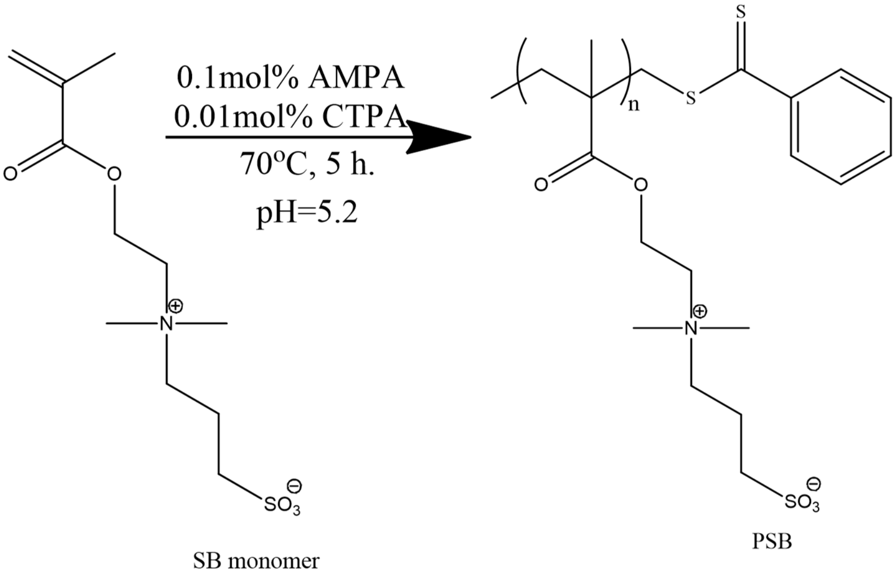

Scheme S2. RAFT polymerization to obtain PSB

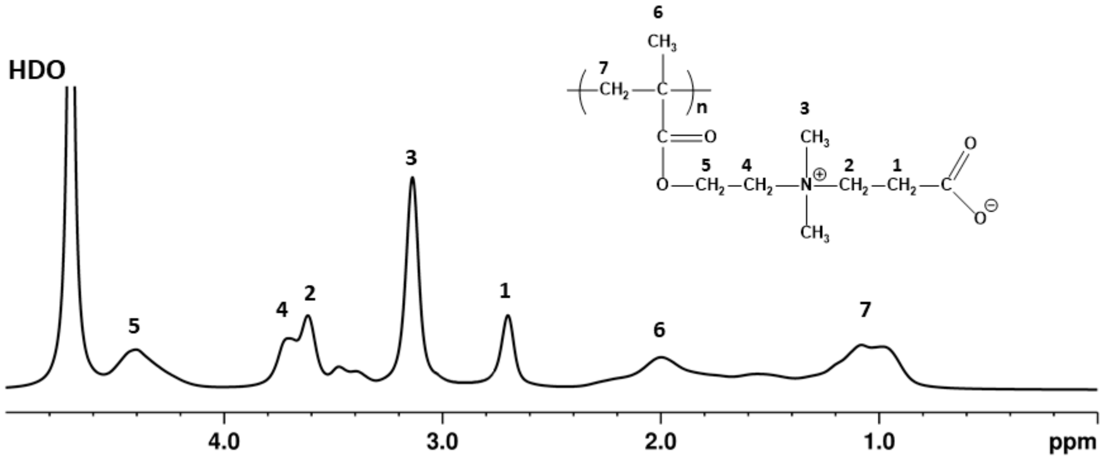

Figure S1. ^{1}H spectrum of PCB in D_2O.

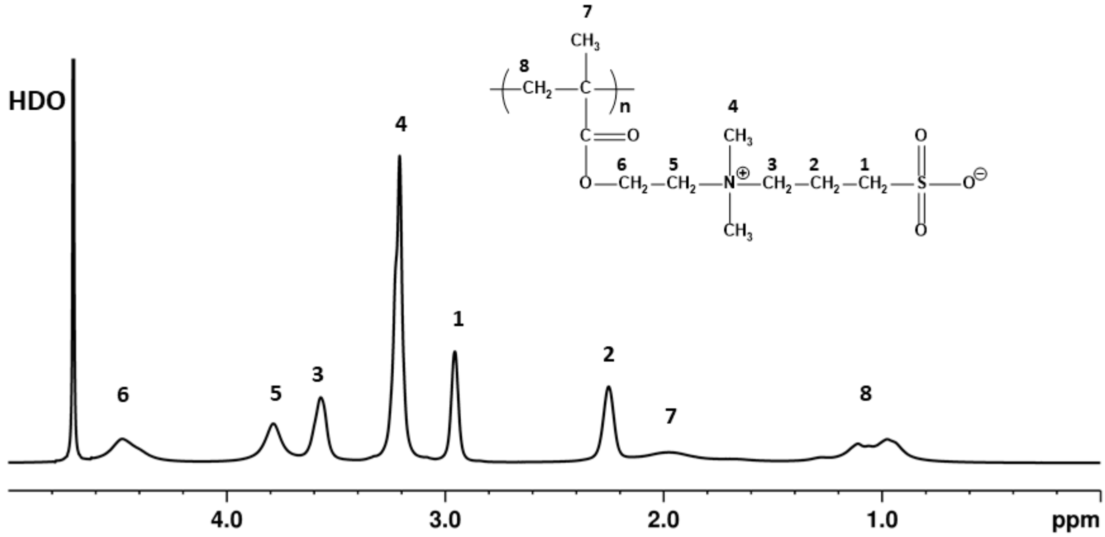

Figure S2. ^{1}H spectrum of PSB in D_2O.

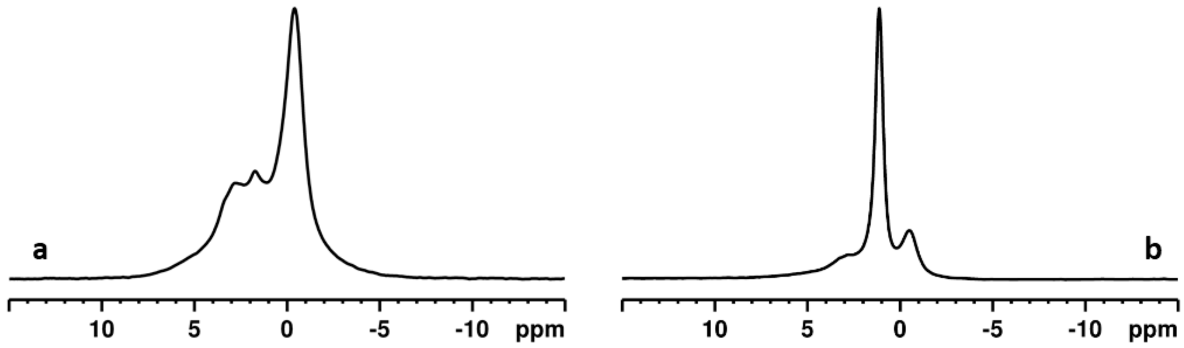

Figure S3. $^{1}H \rightarrow ^{31}P$ CP-MAS spectra of: a) pure A-CaP and b) the hybrid A-CaP/PSB materials from series A.

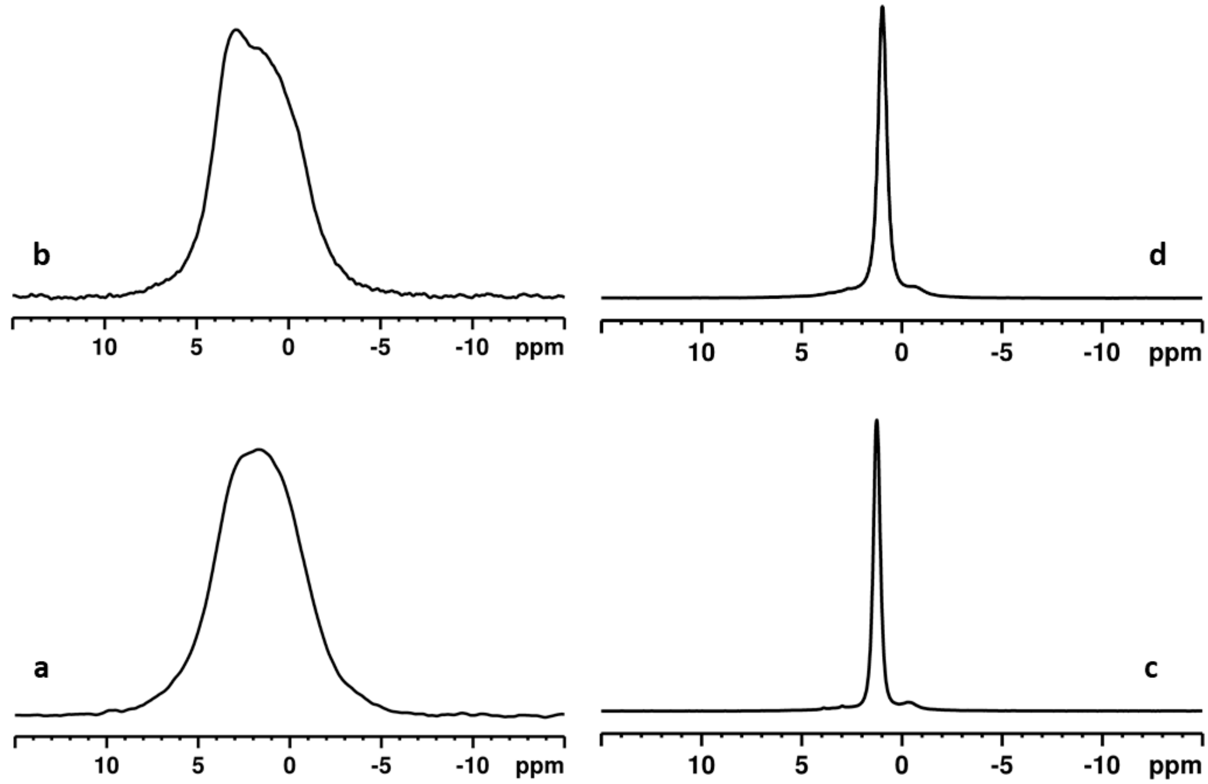

Figure S4. $^{1}H \rightarrow ^{31}P$ CP-MAS spectra of: a) hybrid B-CaP/PCB material measured at mixing time of 100 μs, b) hybrid B-CaP/PCB material measured at mixing time of 4 ms, c) pure B-CaP and d) the hybrid B-CaP/PSB materials from series B.
